# Does glycemic control reverse dispersion of ventricular repolarization in type 2 diabetes?

**DOI:** 10.1186/s12933-014-0125-8

**Published:** 2014-08-21

**Authors:** Takayuki Miki, Toshiyuki Tobisawa, Tatsuya Sato, Masaya Tanno, Toshiyuki Yano, Hiroshi Akasaka, Atsushi Kuno, Makoto Ogasawara, Hiromichi Murase, Shigeyuki Saitoh, Tetsuji Miura

**Affiliations:** Department of Cardiovascular, Renal and Metabolic Medicine, Sapporo Medical University School of Medicine, South-1 West-16, Chuo-ku Sapporo, 060-8543 Japan; Department of Pharmacology, Sapporo Medical University School of Medicine, Sapporo, Japan; Department of Nursing, Sapporo Medical University School of Health Sciences, Sapporo, Japan

**Keywords:** Type 2 diabetes, Glycemic control, QT dispersion, Ventricular repolarization

## Abstract

**Background:**

Abnormal ventricular repolarization is a predictor of cardiovascular mortality. In this study, we tested the hypothesis that glycemic control reverses abnormal ventricular repolarization in patients with type 2 diabetes.

**Methods:**

We analyzed longitudinal changes in repolarization indices of electrocardiograms in retrospectively enrolled 44 patients with type 2 diabetes and 44 age-matched healthy subjects.

**Results:**

In the diabetic group, BMI was greater, levels of HbA1c (10.0 ± 1.6 vs. 5.6 ± 0.3%) and triglyceride were higher and level of HDL cholesterol was lower than those in the control group. Although mean QTc intervals were similar (413.6 ± 18.5 vs. 408.3 ± 22.7 ms), QT dispersion (41.8 ± 15.4 vs. 28.7 ± 7.7 ms) and Tpeak-Tend in lead V5 (83.6 ± 13.6 vs. 71.3 ± 10.3 ms) were significantly longer in the diabetic group than in the control group, indicating increased heterogeneity of ventricular repolarization in type 2 diabetes. During follow-up of 36 patients in the diabetic group for 787 ± 301 days, HbA1c level decreased to 7.3 ± 1.6%, while BMI did not significantly change. In contrast to HbA1c, QT dispersion (45.8 ± 15.0 ms) and Tpeak-Tend in lead V5 (83.6 ± 10.6 ms) were not significantly reduced during the follow-up period. There was no correlation between the change in HbA1c and the change in QT dispersion or Tpeak-Tend.

**Conclusions:**

Increased heterogeneity of ventricular repolarization in type 2 diabetic patients was not reduced during the relatively short follow-up period despite significantly improved glycemic control.

## Background

The number of patients with type 2 diabetes has been increasing worldwide in the past two decades, and these patients are predisposed to serious cardiovascular morbidity and mortality [1,2]. Despite recent progress in coronary intervention strategies, diabetes is associated with high mortality after acute myocardial infarction (MI) due to extensive atherosclerotic lesions and also a hypertrophied and dysfunctional left ventricle [3]. It has been reported that post-MI patients with diabetes have higher incidences of heart failure, recurrent myocardial ischemic events and sudden cardiac death (SCD) than do those without diabetes [4]. In the UKPDS, glycemic control significantly reduced the incidence of microvascular disease but had limited effects on cardiovascular events including SCD [5]. Furthermore, recent large clinical trials have shown that no significant reduction of major adverse cardiac events was achieved by 2–5 years of intensive glycemic control [6–10].

An electrocardiogram (ECG) is the most widely used noninvasive diagnostic test for cardiovascular risk stratification. It is well known that repolarization abnormalities, such as prolonged QT interval (or heart rate-corrected QT interval (QTc)) and increased QT dispersion, are associated with increased risk of malignant ventricular arrhythmias and SCD in high-risk populations (i.e., patients with myocardial infarction and cardiomyopathy) [11]. Furthermore, most, but not all, studies have shown that prolonged QTc and increased QT dispersion were predictors of all-cause and cardiovascular mortality in the general population [12,13] and probably in diabetic patients as well [14–17]. The interval from the peak to the end of the T wave (Tpeak-Tend) is known to reflect transmural repolarization heterogeneity and has been associated with increased risk of mortality not only in high-risk patients [18,19] but also in the general population [20,21]. Importantly, Tpeak-Tend has been shown to predict cardiovascular mortality even when the QTc interval is normal [21]. However, its importance in diabetic patients has yet to be determined. In addition, it is unclear whether blood glucose-lowering therapy modifies the spatial heterogeneity in repolarization, if any, in diabetic patients. In the present study, we tested the hypotheses that repolarization heterogeneity is enlarged in patients with type 2 diabetes and that glycemic control alleviates the abnormality in repolarization.

## Methods

This study was conducted in strict adherence with the principles of the Declaration of Helsinki and was approved by the Clinical Investigation Ethics Committee of Sapporo Medical University Hospital.

### Subjects

**Figure 1:**
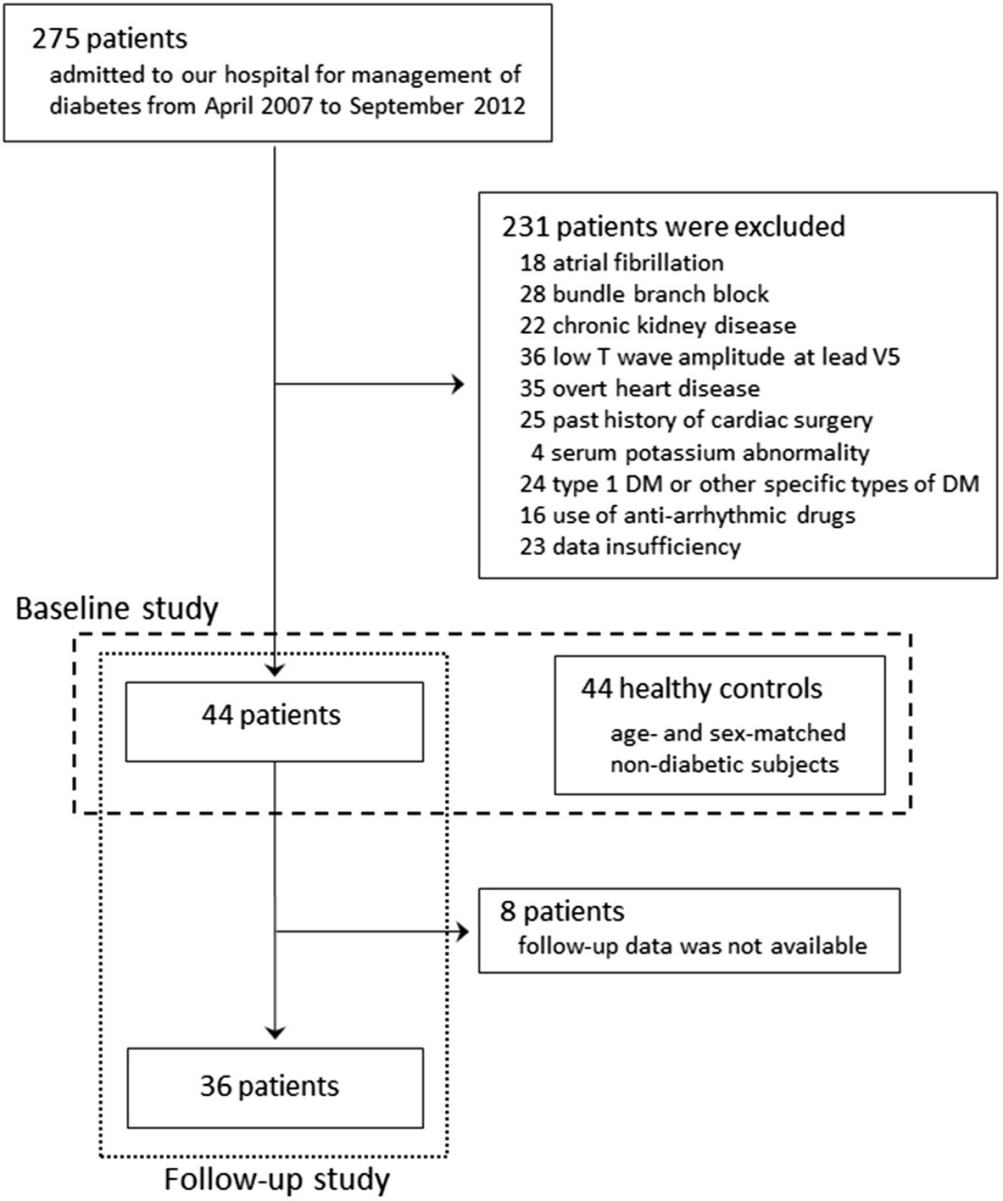
**Enrollment and follow-up of study participants.**

We retrospectively analyzed data for 275 consecutive patients who were admitted to our hospital for management of type 2 diabetes from April 2007 to September 2012. Control subjects were chosen from the Tanno-Sobetsu cohort [22], in which residents of two Japanese rural towns, Tanno and Sobetsu, have been prospectively followed up by annual or biannual medical examination, including standard blood tests and ECG. We selected age- and sex-matched healthy subjects who had not been receiving any medications. Exclusion criteria were type 1 diabetes and other specific types of diabetes, atrial fibrillation, past history of cardiac surgery, chronic kidney disease at stage 4 or higher, serum potassium abnormality (<3.5 or >5.5 mEq/l), use of an anti-arrhythmic drug (except a β blocker used for hypertension or coronary artery disease), bundle branch block, overt heart disease, and low T wave amplitude at lead V5 (<0.1 mV). By the exclusion criteria, 231 diabetic patients were excluded, and 44 type 2 diabetic patients and 44 age- and sex-matched non-diabetic controls were enrolled in this study (Figure 1). Autonomic neuropathy was defined as a loss of heart rate variability or postural hypotension with a fall in systolic blood pressure ≥20 mmHg [23,24]. After discharge from our hospital, the patients were followed up at the outpatient clinic. We retrospectively analyzed longitudinal changes in clinical parameters during follow-up for 227–1374 days (i.e., follow-up until April 2014) under glucose-lowering therapy. The medication was adjusted by each physician’s decision to optimize the glycemic control.

Clinical characteristics, laboratory data and ECG findings were compared between the type 2 diabetic group and the control group and before and after the glycemic control within the type 2 diabetic group.

### ECG recordings and measurements

Standard resting 12-lead ECGs were recorded at 25 mm/s paper speed and 10 mm/mV amplitude. The beginning of the QT interval was defined as first deflection of the QRS complex. The end of the T wave was defined as the intersection of the tangent to the down slope of the T wave and the isoelectric line when not followed by a U wave or if distinct from the following U wave. If a U wave followed by the T wave, T wave offset was measured as the nadir between the T and U waves. In case of a flat T wave or TU merge without nadir, the end of the T wave could not be determined and the lead was excluded from measurements. The QT intervals were measured in all 12 leads and corrected for heart rate (QTc) by Bazett’s formula, together with a sex-specific method described by Rautaharju and Zhang [25] according to AHA/ACCF/HRS guidelines [26]. QT dispersion and QTc dispersion were calculated as the difference between the maximum and minimum QT interval and QTc interval, respectively, among the 12 leads. Tpeak-Tend was defined as the interval from the peak of positive T wave and the end of the T wave and was measured in lead V5.

### Statistical analysis

Numeric variables are expressed as means ± SD. Differences between two groups were tested by Student’s *t*-test. Relationships between parameters were examined by the use of simple linear regression analyses. Multiple regression analyses were performed to determine the relationships between clinical parameters, QT dispersion and Tpeak-Tend. Changes in clinical and electrocardiographic variables and medication during follow-up periods within the type 2 diabetes group (36 patients) were compared by the paired *t*-test and Fisher’s exact test, respectively. Statistical analyses were carried out using JMP (version11 SAS Institute, Cary, NC, USA). To calculate statistical power for differences in QT dispersion and Tpeak-Tend, we used “Power and Sample Size Calculation version 3.0.43, 2011”. All statistical tests were two-tailed and differences were considered to be statistically significant if p was less than 0.05.

## Results

### Baseline characteristics

Clinical characteristics in the type 2 diabetes and control groups are shown in Table 1. Body mass index (BMI) was larger and systolic blood pressure (BP) was higher in the type 2 diabetes group than in the control group. However, BP was relatively well-controlled by medications in most of the diabetic patients (124.8 ± 16.8/74.2 ± 9.4 mmHg), with only 8 patients (18%) showing systolic BP ≥140 mmHg. Duration of diabetes was 12.5 ± 12.0 years (median, 6.5 years) and 72% of the patients had one or more complications: 14 with retinopathy, 18 with nephropathy (stage 2 or 3), 7 with autonomic neuropathy, and 9 with coronary artery disease. Autonomic neuropathy in the seven patients was diagnosed from postural hypotension (n = 2), reduced coefficient of variance of RR intervals (CV_RR_) on ECG (n = 4) or diabetic neurogenic bladder (n = 1). Patients with coronary artery disease had been medically treated with or without prior coronary interventions, and none of them showed myocardial ischemia in exercise ECG tests or stress myocardial scintigraphy at the time of study enrollment. Glycemic control was poor (fasting plasma glucose: 170.3 ± 49.5 mg/dl, HbA1c: 10.0 ± 1.6%) at the time of admission. As expected in poorly controlled diabetes, triglyceride level was significantly higher and high-density lipoprotein cholesterol (HDL-C) level was significantly lower in type 2 diabetic patients than in controls. Low-density lipoprotein cholesterol (LDL-C) levels were comparable in the two groups, most likely as a result of cholesterol-lowering therapy in 36% of the patients, mainly with a statin.

**Table 1:** **Baseline characteristics**

	**Type 2 diabetes**	**Control**	**P**
	**(n = 44)**	**(n = 44)**	
***Clinical variables***			
Age (years)	60.6 ± 13.8	58.4 ± 11.4	0.353
Male	24 (54.5%)	22 (50.0%)	0.674
BMI (kg/m^2^)	26.7 ± 4.4	22.6 ± 2.8	<0.001
SBP (mmHg)	124.8 ± 16.8	118.3 ± 13.4	0.048
DBP (mmHg)	74.2 ± 9.4	71.6 ± 9.7	0.204
Smoking	24 (54.5%)	20 (45.5%)	0.400
Duration of DM (years)	12.5 ± 12.0	N/A	
Retinopathy	14 (31.8%)	N/A	
Nephropathy	18 (40.9%)	N/A	
Autonomic neuropathy	7 (15.9%)	N/A	
CAD	9 (20.5%)	N/A	
***Laboratory variables***			
FPG (mg/dl)	170.3 ± 49.5	89.7 ± 8.0	<0.001
HbA1c (%)	10.0 ± 1.6	5.6 ± 0.3	<0.001
Triglyceride (mg/dl)	228.1 ± 246.0	88.2 ± 38.9	<0.001
HDL-C (mg/dl)	44.5 ± 12.4	54.8 ± 10.2	<0.001
LDL-C (mg/dl)	115.9 ± 40.0	129.4 ± 27.4	0.0069
Creatinine (mg/dl)	0.66 ± 0.22	0.64 ± 0.14	0.597
Uric acid (mg/dl)	5.0 ± 1.4	5.1 ± 1.4	0.596
Potassium (mEq/l)	4.1 ± 0.4	4.3 ± 0.1	0.074
***Medications***			
ACE-I/ARB	16 (36.4%)	N/A	
CCB	13 (29.5%)	N/A	
β blocker	5 (11.4%)	N/A	
Other antihypertensive drugs	8 (18.2%)	N/A	
Sulphonylurea	19 (43.2%)	N/A	
α-glucosidase inhibitor	19 (43.2%)	N/A	
Biguanide	16 (36.4%)	N/A	
DPP-4 inhibitor	14 (31.8%)	N/A	
Insulin	8 (18.2%)	N/A	
Other antidiabetic drugs	6 (13.6%)	N/A	
Statin	14 (31.8%)	N/A	
Fibrate	2 (4.5%)	N/A	
***Electrocardiographic variables***			
Heart rate (bpm)	74.2 ± 15.5	61.0 ± 8.6	<0.001
V1S + V5R (mV)	2.37 ± 0.58	2.14 ± 0.68	0.093
QTc mean (ms)	413.6 ± 18.5	408.3 ± 22.7	0.229
QT dispersion (ms)	41.8 ± 15.4	28.7 ± 7.7	<0.001
QTc dispersion (ms)	45.9 ± 16.3	28.8 ± 7.3	<0.001
Tpeak-Tend in V5 (ms)	83.6 ± 13.6	71.3 ± 10.3	<0.001

BMI = body mass index, SBP = systolic blood pressure, DBP = diastolic blood pressure, CAD = coronary artery disease, FPG = fasting plasma glucose, HbA1c = glycated hemoglobin, HDL-C = high-density lipoprotein cholesterol, LDL-C = low-density lipoprotein cholesterol, ACE-I = angiotensin-converting enzyme inhibitor, ARB = angiotensin II receptor blocker, CCB = calcium channel blocker, DPP-4 inhibitor = dipeptidyl peptidase-4 inhibitor. QTc = corrected QT (by Bazett’s formula), N/A= not applicable. Values are means ± SD or absolute numbers (frequency percentages).

### Electrocardiographic measurements

Heart rate was significantly higher in type 2 diabetic patients than in controls (Table 1). The sum of S wave depth in lead V1 and R wave height in lead V5 (V1S + V5R, an index for left ventricular mass, with ≥3.5 mV being defined as left ventricular hypertrophy by the Sokolow-Lyon voltage criterion) tended to be larger in diabetic patients, although only two subjects in each group met the ECG criterion for left ventricular hypertrophy. Mean QTc interval (413.6 ± 18.5 vs. 408.3 ± 22.7 ms) was not significantly longer in type 2 diabetic patients than in controls, and a similar trend was observed when QT was adjusted using the method of Rautaharju and Zhang (407.4 ± 15.6 vs. 406.9 ± 21.0 ms). In contrast, QT dispersion (41.8 ± 15.4 vs. 28.7 ± 7.7 ms) and QTc dispersion (45.9 ± 16.3 vs. 28.8 ± 7.3 ms) were significantly increased in type 2 diabetic patients compared with those in controls. Increased QTc dispersion in type 2 diabetic patients was also detected by use of the correction by Rautaharju and Zhang [25] (44.9 ± 15.5 vs. 35.1 ± 12.8 ms, p < 0.01). Tpeak-Tend (83.6 ± 13.6 vs. 71.3 ± 10.3 ms) and Tpeak-Tend/QT ratio (0.220 ± 0.028 vs. 0.175 ± 0.022) were significantly longer in the diabetic group than in the control group, indicating increased heterogeneity of ventricular repolarization in type 2 diabetes.

**Figure 2:**
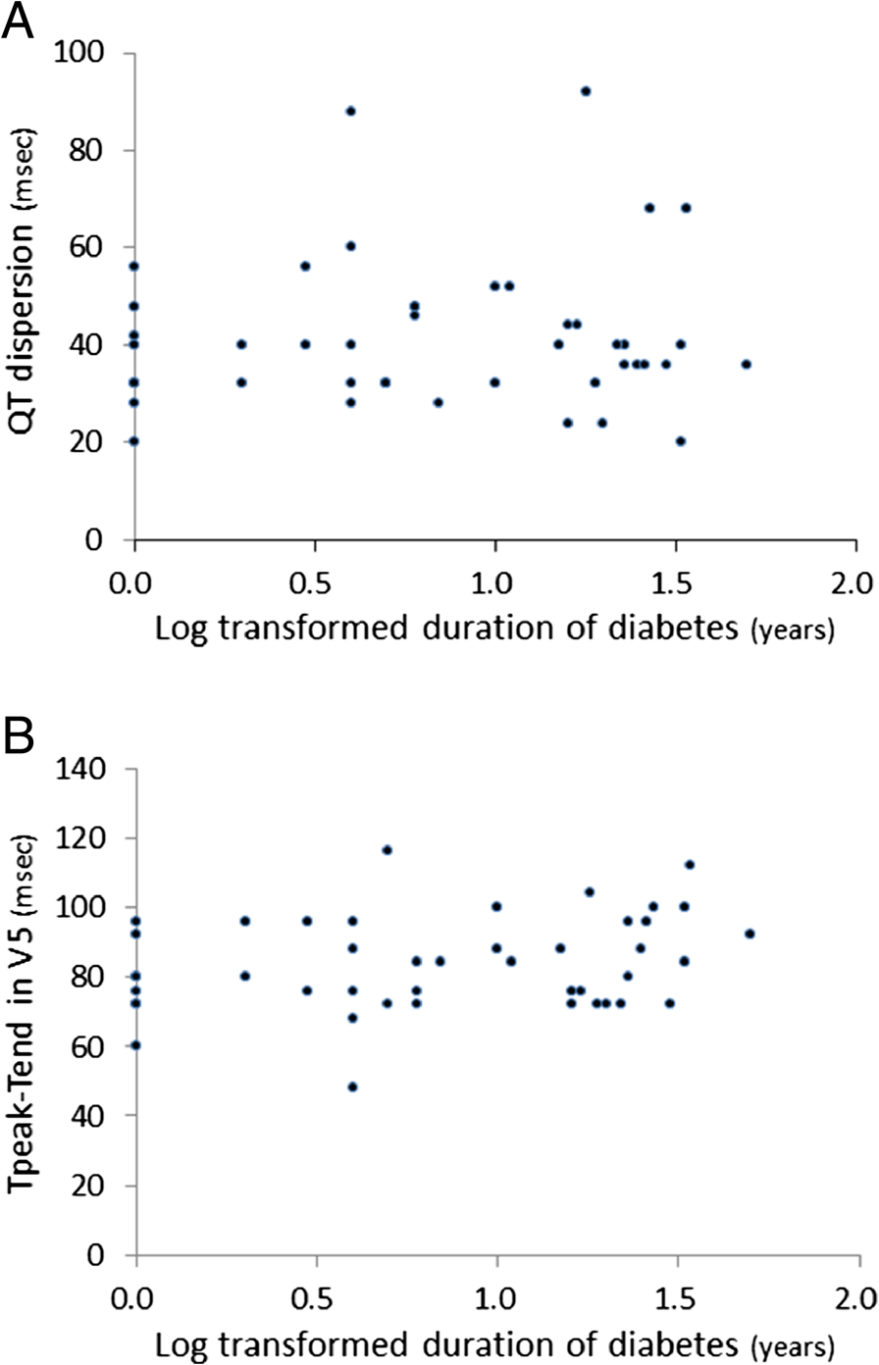
**Neither QT dispersion (A) nor Tpeak-Tend (B) was correlated with duration of diabetes (log transformed).**

Multiple regression analysis indicated that HbA1c and systolic BP were independent determinants of both QT dispersion and Tpeak-Tend, indices of heterogeneity in ventricular repolarization (Table 2). On the other hand, neither QT dispersion nor Tpeak-Tend in type 2 diabetes was correlated with duration of diabetes (Figure 2).

**Table 2:** **Multiple regression analyses for electrical heterogeneity indices**

		**QT dispersion**					**Tpeak-Tend**			
	**B**	**SE**	**β**	**t**	**p**	**B**	**SE**	**β**	**t**	**p**
Age (years)	0.043	0.120	0.039	0.353	0.720	−0.001	0.110	−0.001	−0.008	0.990
Sex (male)	−0.148	1.400	−0.011	−0.109	0.910	−1.520	1.300	−0.113	−1.180	0.240
BMI (kg/m^2^)	−0.093	0.370	−0.028	−0.248	0.810	−0.295	0.350	−0.092	−0.838	0.400
SBP (mmHg)	0.233	0.095	0.261	2.450	0.016	0.188	0.090	0.216	2.100	0.039
HbA1c (%)	2.050	0.660	0.374	3.130	0.002	2.710	0.620	0.504	4.380	<0.001
Triglyceride (mg/dl)	−0.009	0.008	−0.126	−1.140	0.260	−0.012	0.008	−0.166	−1.560	0.120
	n = 88, R^2^ = 0.224, AIC = 706.0					n = 88, R^2^ = 0.281, AIC = 695.5				

BMI = body mass index, SBP = systolic blood pressure, HbA1c = glycated hemoglobin.

### Effect of glycemic control on heterogeneity in ventricular repolarization

After discharge from our hospital, 31 patients were followed at the out-patient clinic of our hospital and enrolled in the follow-up study. The remaining 13 patients were followed at affiliated clinics. We confirmed that all of the 13 patients have been free from cardiovascular events, but ECG and laboratory data of 8 patients were not available for the present analyses. Therefore, we analyzed changes in electrocardiographic variables and clinical data for 36 patients at baseline and during follow-up after treatment of diabetes (Table 3, Figure 1). The proportions of patients on each medication did not significantly change during a mean follow-up period of 787.0 ± 300.8 days (median, 754 days), except for increased frequency in use of dipeptidyl peptidase (DPP)-4 inhibitors at the end of the follow-up period. HbA1c level was significantly reduced from 10.0 ± 1.7 to 7.3 ± 1.6%, although BMI and BP were unchanged during the follow-up period. Triglyceride and LDL-C were significantly reduced after treatments, probably due to low calorie/fat intake as diet therapy, improvement of glycemic control, increased use of statins (50.0 vs 33.3%) and/or their combination. Serum creatinine level significantly increased from 0.67 ± 0.23 to 0.99 ± 0.85 mg/dl, and this trend was still observed even after exclusion of two patients who developed stage 5 nephropathy (0.80 ± 0.28 mg/dl).

**Table 3:** **Changes in parameters in type 2 diabetic patients (n = 36)**

	**Baseline**	**Follow-up**	**P**
***Clinical variables***			
Duration of treatments (days)	-	787.0 ± 300.8	
BMI (kg/m^2^)	26.5 ± 4.7	26.0 ± 4.8	0.239
SBP (mmHg)	126.6 ± 18.0	127.5 ± 14.9	0.564
DBP (mmHg)	75.1 ± 9.9	71.0 ± 9.4	0.051
***Laboratory variables***			
HbA1c (%)	10.0 ± 1.7	7.3 ± 1.6	<0.001
Triglyceride (mg/dl)	184.8 ± 120.2	148.5 ± 81.9	0.043
HDL-C (mg/dl)	44.4 ± 11.8	46.9 ± 13.1	0.289
LDL-C (mg/dl)	116.0 ± 41.5	97.3 ± 28.8	0.020
Creatinine (mg/dl)	0.67 ± 0.23	0.99 ± 0.85	0.011
Uric acid (mg/dl)	4.9 ± 1.2	5.1 ± 1.4	0.504
Potassium (mEq/l)	4.2 ± 0.4	4.1 ± 0.5	0.734
***Medications***			
ACE-I/ARB	13 (36.1%)	18 (50.0%)	0.341
CCB	11 (30.6%)	12 (33.3%)	1.000
β blocker	5 (13.9%)	6 (16.7%)	1.000
Other antihypertensive drugs	6 (16.7%)	8 (22.2%)	0.767
Sulphonylurea	17 (47.2%)	13 (36.1%)	0.474
α-glucosidase inhibitor	16 (44.4%)	11 (30.6%)	0.330
Biguanide	14 (38.9%)	18 (50.0%)	0.477
DPP-4 inhibitor	10 (27.8%)	27 (75.0%)	<0.001
Insulin	7 (19.4%)	10 (27.8%)	0.580
Other antidiabetic drugs	4 (11.1%)	2 (5.6%)	0.674
Statin	12 (33.3%)	18 (50.0%)	0.232
Fibrate	2 (5.6%)	1 (2.8%)	1.000
***Electrocardiographic variables***			
Heart rate (bpm)	73.6 ± 14.4	70.6 ± 11.0	0.156
V1S + V5R (mV)	2.42 ± 0.60	2.58 ± 0.74	0.097
QTc mean (ms)	414.1 ± 19.3	414.8 ± 23.0	0.843
QT dispersion (ms)	41.4 ± 16.6	45.8 ± 15.0	0.165
QTc dispersion (ms)	45.1 ± 16.8	46.8 ± 14.4	0.564
Tpeak-Tend in V5 (ms)	85.0 ± 12.9	83.6 ± 10.6	0.563

BMI = body mass index, SBP = systolic blood pressure, DBP = diastolic blood pressure, CAD = coronary artery disease, FPG = fasting plasma glucose, HbA1c = glycated hemoglobin, HDL-C = high-density lipoprotein cholesterol, LDL-C = low-density lipoprotein cholesterol, ACE-I = angiotensin-converting enzyme inhibitor, ARB = angiotensin II receptor blocker, CCB = calcium channel blocker, DPP-4 inhibitor = dipeptidyl peptidase-4 inhibitor, QTc = corrected QT (by Bazett’s formula), Values are means ± SD or absolute numbers (frequency percentages).

**Figure 3:**
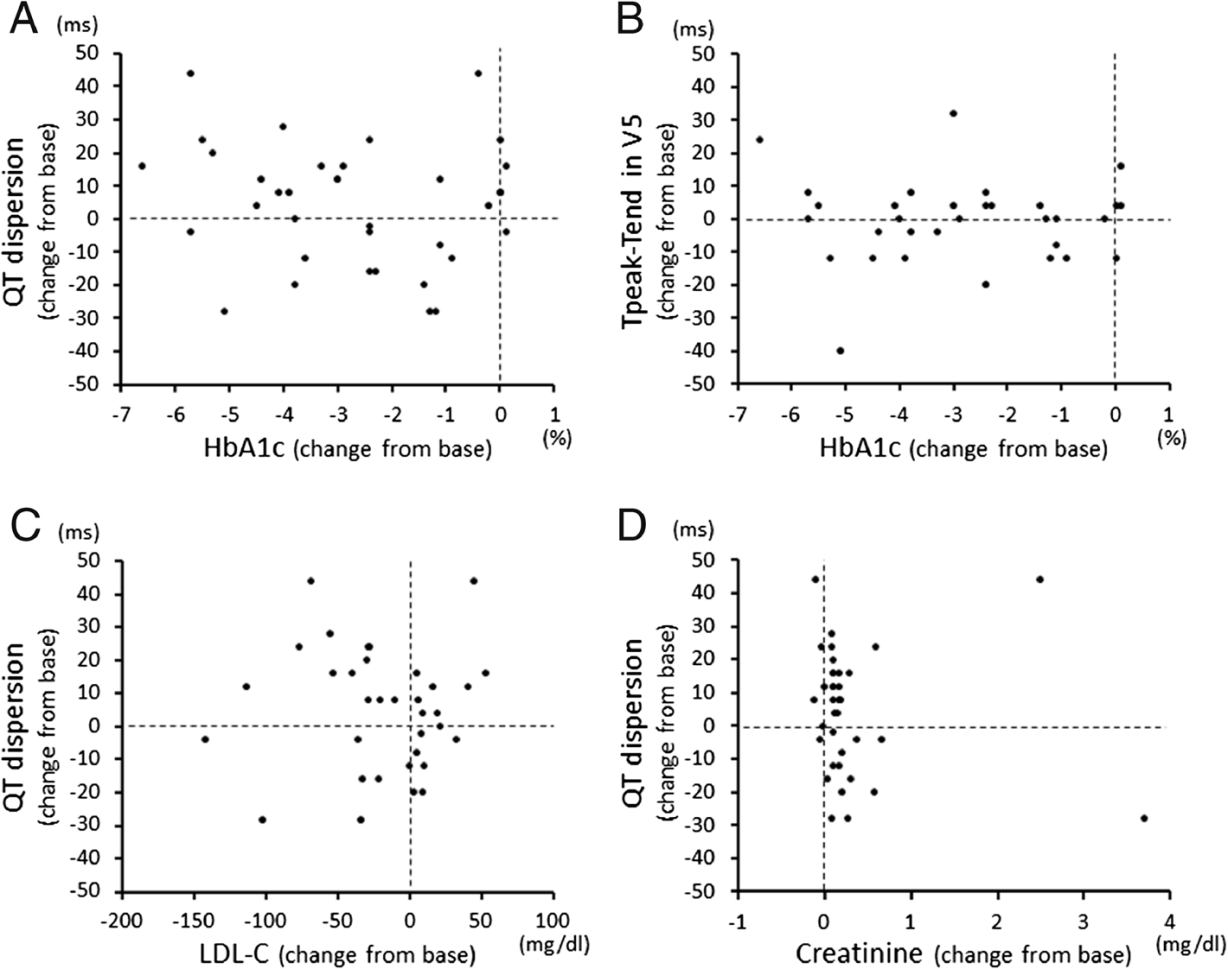
**There were no correlations between changes in QT dispersion (A) and Tpeak-Tend (B) and change in HbA1c level.** Change in QT dispersion was also not correlated with change in LDL-C level **(C)** or creatinine level **(D)**.

Heart rate and voltage index in ECG were not different before and after glycemic control. Despite the considerable reduction in HbA1c after treatment of diabetes, mean QTc interval, QT dispersion and Tpeak-Tend in lead V5 did not significantly changed (Table 3). Furthermore, there was no significant correlation between change in HbA1c, creatinine or LDL-C and change in QT dispersion or Tpeak-Tend during the follow-up period (Figure 3).

## Discussion

The majority of previous studies have shown that QTc interval was prolonged in diabetic patients compared to that in non-diabetic controls [27,28]. In the present study, however, QTc interval in type 2 diabetic patients (413.6 ± 18.5 ms) was similar to that in control subjects (408.3 ± 22.7 ms). Although Bazett’s formula has been the most frequently used method to adjust QT intervals for heart rate, it tends to underestimate or overestimate the duration of repolarization when heart rate is relatively slow or fast. Since heart rate was faster in diabetic patients than in control subjects, we also corrected QT intervals for heart rate using a method proposed by Rautaharju and Zhang [25]. However, as was the case with Bazett’s formula, the method of Rautaharju and Zhang also did not yield a significant difference in QTc interval between diabetic patients and controls (407.4 ± 15.6 vs. 406.9 ± 21.0 ms). In contrast, QT dispersion and QTc dispersion, indices for global dispersion, and Tpeak-Tend, an index for transmural dispersion, were significantly increased in type 2 diabetic patients compared to those in controls. It has been shown that QT dispersion and/or QTc dispersion are better prognostic markers than QTc interval in diabetes to predict cardiovascular mortality [16,17]. The present results suggest that increases in QT dispersion, QTc dispersion and Tpeak-Tend precede the QTc prolongation, thereby being useful for detection of repolarization abnormality at an earlier stage. Alternatively, QT dispersion, QTc dispersion and Tpeak-Tend may be more sensitive than QTc interval for detecting even slight abnormalities in repolarization.

Electrical repolarization abnormalities have been shown to be associated with increased systolic BP, left ventricular hypertrophy, presence of coronary artery disease, autonomic dysfunction or microalbuminuria in patients with diabetes [24,27,29,30]. Since these complications are increased when the duration of diabetes is prolonged, we presumed that repolarization abnormality would be related to disease duration. However, neither QT dispersion nor Tpeak-Tend was correlated with duration of diabetes (Figure 2). This is consistent with the results of a study by Festa et al. [27] showing that QT interval was already prolonged in newly diagnosed diabetes. We also examined the relationship between presence of coronary artery disease or autonomic dysfunction and repolarization abnormalities. However, the values of both QT dispersion and Tpeak-Tend were similar in diabetic patients regardless of the presence or absence of coronary artery disease or autonomic dysfunction (data not shown). There is a possibility that the small number of patients with these complications made it difficult to detect the difference, but our results suggest that the presence of coronary artery disease and autonomic dysfunction are not major predictors of repolarization abnormalities.

We found that HbA1c was an independent and strong explanatory variable for increased QT dispersion and Tpeak-Tend in this study (Table 2). This result is in accordance with results of studies showing that poor glycemic control was associated with prolonged QT interval [31,32]. However, except for the association with HbA1c and BP, relationships between diabetes-related changes in clinical parameters and repolarization abnormality have not been clarified in previous studies [27,28,31,32]. Therefore, we tested the hypothesis that glycemic control could improve repolarization abnormality in diabetic patients. While HbA1c level significantly decreased from 10.0 ± 1.7 to 7.3 ± 1.6% during the follow-up period, none of the repolarization indices improved after treatment of diabetes. Furthermore, there was no significant correlation between change in HbA1c and change in QT dispersion or Tpeak-Tend during the follow-up period (Figure 3). To the best of our knowledge, this is the first report that glycemic control failed to improve repolarization abnormalities.

Several classes of anti-diabetic drugs were used for glycemic control in the present study subjects. Previous studies have shown that biguanide, DPP-4 inhibitors and sodium glucose cotransporter 2 inhibitors do not modify ventricular repolarization [33–35]. On the other hand, sulfonylurea inhibits ATP-sensitive K^+^ channels not only in pancreatic β cells but also on the sarcolemma of cardiomyocytes, resulting in prolongation of the QT interval [33]. Although approximately 40% of the patients were treated with sulfonylurea in the present study, those patients were taking glimepiride, which has less effect than glibenclamide on cardiac ATP-sensitive K^+^ channels [36]. Furthermore, the number of patients treated with sulfonylurea was not increased during the follow-up. Therefore, it is unlikely that the failure of glycemic control to improve repolarization abnormalities is attributable to the medicines used for glycemic control in the present study. Recent large clinical trials have shown that intensive glycemic control failed to reduce cardiovascular and all-cause mortality [6–10] during 2 to 5 years of treatment. A benefit of glycemic control in reducing the risk of cardiovascular disease was observed only when the follow-up period was long (10–20 years) in even newly diagnosed diabetic patients [37]. There is the possibility that the follow-up period (787.0 ± 300.8 days) in the present study was too short to show alleviation of the repolarization abnormality by tight glycemic control. Nevertheless, the present results are consistent with the results of recent clinical trials showing that intensive glycemic control failed to reduce cardiovascular mortality [6–10].

In contrast to the failure of glycemic control to improve repolarization abnormalities in the present study, protective effects of BP and lipid control have been reported in patients with diabetes and/or hypertension [28,38,39]. Treatment with an angiotensin-converting enzyme inhibitor and a calcium channel blocker significantly decreased QT dispersion in patients with hypertension, and this effect was correlated with the degree of left ventricular hypertrophy [38]. In hypertensive patients with diabetes, treatment with aliskiren, a direct renin inhibitor, reduced QT dispersion at 12 weeks after treatment [39]. These results may reflect the outcomes of clinical trials showing that interventions for hypertension and dyslipidemia have improved cardiovascular and all-cause mortality in patients with diabetes [40,41]. Festa et al. [27] showed that systolic BP and LV mass, but not glucose level, were determinants of the QT interval in diabetic patients. Cox et al. [15] reported that systolic BP was higher in a prolonged QTc group (152.8 mmHg) than that in a normal QTc group (139.6 mmHg) of type 2 diabetic patients, though HbA1c levels in the two groups were similar (8.2% vs. 7.7%). In the present study, multiple regression analysis revealed that systolic BP was an independent predictor of QT dispersion and Tpeak-Tend (Table 2), and an ECG marker of left ventricular mass (V1S + V5R) tended to be higher in diabetic patients. These results suggested that high BP and consequent increase in ventricular mass are stronger determinants than HbA1c for increased heterogeneity of ventricular repolarization in diabetic patients. In the present study, BP in diabetic patients was well-controlled by medications both at baseline and during the follow-up periods (Table 3), indicating that significant improvement of glycemic control does not attenuate repolarization abnormality by diabetes even under good BP control.

Treatment with a statin has been shown to improve repolarization heterogeneity in patients with diabetes in a study by Tekin et al. [28]. They reported that treatment of diabetic patients with simvastatin for 12 weeks decreased LDL-C from 142 mg/dl to 80 mg/dl and reduced QT and QTc dispersions by 24% and 27%, respectively. Whether the LDL-C-reducing property of simvastatin or its pleiotropic effect contributed to the improvement of repolarization heterogeneity remains unclear. In the present study, QT dispersion was not reduced during the follow-up period, although LDL-C was reduced by 17% in association with an increase in the proportion of patients on statins (Table 3). A plausible explanation for the discrepancy between the present results and those in the study by Tekin et al. [28] is well-controlled LDL-C level at baseline in the present study: LDL-C level was within the recommendation range (LDL-C <120 mg/dl in diabetic patients, Japan Atherosclerosis Society Guidelines for Prevention of Atherosclerotic Cardiovascular Diseases 2012) at baseline and change in LDL-C level during the follow-up period was within normal ranges.

It has been shown that severe hypoglycemia is associated with increased cardiovascular mortality, and fatal arrhythmias caused by abnormal ventricular repolarization during hypoglycemia could be one of the mechanisms [42,43]. Significant prolongation of QT interval during hypoglycemia has also been observed in previous studies [42–45]. In this study, there was no episode of severe hypoglycemia in patients during hospitalization or the follow-up, but mild hypoglycemia and hypoglycemia unawareness could not be totally excluded in retrospective analysis of medical records. Hence, to examine the possibility that mild hypoglycemic episodes, if any, had an impact on ventricular repolarization in the diabetic patients, we divided the diabetic patients into a subgroup treated with sulfonylurea and/or insulin and a subgroup treated with other agents. Although sulfonylurea and insulin are known to increase the risk of hypoglycemia compared with other agents, QT dispersion and Tpeak-Tend were similar in the subgroups of patients treated with or without sulfonylurea and/or insulin (39.5 ± 15.0 vs. 43.8 ± 15.8 ms for QT dispersion and 84.6 ± 13.0 vs. 82.8 ± 14.2 ms for Tpeak-Tend). We also analyzed blood glucose levels at the outpatient clinic when follow-up ECG was taken in the 36 diabetic patients. Glucose levels ranged from 80 to 280 mg/dl (mean: 156.3 ± 44.3 mg/dl), and thus none of follow-up ECGs were recorded at the time of hypoglycemia. These findings argue against the possibility that improvement of ventricular repolarization by glycemic control was masked by mild ~ modest hypoglycemic episodes in the present study.

There are several limitations in this study. First, the number of patients was small and the follow-up period of diabetic treatment was relatively short. Statistical power for detection of differences in QT dispersion and Tpeak-Tend between type 2 diabetes and control groups (N = 44 each) was 0.998 and 0.987, respectively, suggesting that it was sufficient power (>0.80) in baseline comparison in this study. On the other hand, statistical power for longitudinal changes in the indices of ventricular repolarization is not large due to the relatively small number of patients (i.e., 0.332 for QT dispersion and 0.094 for Tpeak-Tend), and thus the possibility of a type 2 error cannot be excluded. Second, no major adverse cardiac event or death occurred during the follow-up period. Therefore, the observed impact of increased QT dispersion and Tpeak-Tend on the clinical outcome could not be confirmed.

## Conclusions

Increased heterogeneity of ventricular repolarization in diabetic patients was not normalized by significant glycemic control within a few years. The results are consistent with the results of recent clinical trials showing that intensive glycemic control failed to reduce cardiovascular mortality. Whether intensive glycemic control for more than a few years ultimately reverses the repolarization heterogeneity remains to be further investigated.

### Consent

Informed consent of this retrospective study was obtained from the patient directly or via the study information publicized in the internet.

## Abbreviation

BMI: Body mass index
BP: Blood pressure
DPP-4: Dipeptidyl peptidase-4
ECG: Electrocardiogram
HDL-C: High-density lipoprotein cholesterol
LDL-C: Low-density lipoprotein cholesterol
MI: Myocardial infarction
QTc: Heart rate-corrected QT interval
SCD: Sudden cardiac death
Tpeak-Tend: The interval from the peak to the end of the T wave
